# Use of an electronic medical record dashboard to identify gaps in osteoporosis care

**DOI:** 10.1007/s11657-021-00919-4

**Published:** 2021-04-24

**Authors:** A. Papaioannou, E. McCloskey, A. Bell, D. Ngui, U. Mehan, M. Tan, L. Goldin, A. Langer

**Affiliations:** 1 McMaster University, Hamilton, Ontario Canada; 2 GERAS Centre for Aging Research, St. Peter’s Hospital, Hamilton Health Sciences, 88 Maplewood Ave, Hamilton, Ontario L8M 1W9 Canada; 3 Centre for Metabolic Bone Diseases, University of Sheffield, Sheffield, UK; 4 Department of Family and Community Medicine, University of Toronto, Ontario, Canada; 5 University of British Columbia, Vancouver, British Columbia Canada; 6 Centre for Family Medicine Family Health Team, Kitchener, Ontario Canada; 7 Canadian Centre for Professional Development in Health and Medicine, Toronto, Ontario Canada

**Keywords:** Osteoporosis, Electronic medical record, Decision support tools, Care gaps

## Abstract

**Summary:**

Using an electronic medical record (EMR)-based dashboard, this study explored osteoporosis care gaps in primary care. Eighty-four physicians shared their practice activities related to bone mineral density testing, 10-year fracture risk calculation and treatment for those at high risk. Significant gaps in fracture risk calculation and osteoporosis management were identified.

**Purpose:**

To identify care gaps in osteoporosis management focusing on Canadian clinical practice guidelines (CPG) related to bone mineral density (BMD) testing, 10-year fracture risk calculation and treatment for those at high risk.

**Methods:**

The ADVANTAGE OP EMR tool consists of an interactive algorithm to facilitate assessment and management of fracture risk using CPG. The FRAX® and Canadian Association of Radiologists and Osteoporosis Canada (CAROC) tools were embedded to facilitate 10-year fracture risk calculation. Physicians managed patients as clinically indicated but with EMR reminders of guideline recommendations; participants shared practice level data on management activities after 18-month use of the tool.

**Results:**

Eighty-four physicians (54%) of 154 who agreed to participate in this study shared their aggregate practice activities. Across all practices, there were 171,310 adult patients, 40 years of age and older, of whom 17,214 (10%) were at elevated risk for fracture. Sixty-two percent of patients potentially at elevated risk for fractures did not have BMD testing completed; most common reasons for this were intention to order BMD later (48%), physician belief that BMD was not required (15%) and patient refusal (20%). For patients with BMD completed, fracture risk was calculated in 29%; 19% were at high risk, of whom 37% were not treated with osteoporosis medications as recommended by CPG.

**Conclusion:**

Despite access to CPG and fracture risk calculators through the ADVANTAGE OP EMR tool, significant gaps remain in fracture risk calculation and osteoporosis management. Additional strategies are needed to address this clinical inertia among family physicians.

## Introduction

Osteoporosis is treatable and fractures can be prevented [1]. However, it remains largely undiagnosed and untreated [2–4]. Fewer than 20% of women and 10% of men experiencing a fragility fracture receive therapies to prevent further fractures [2, 3, 5]. Canadian clinical practice guidelines (CPGs) for the assessment and management of osteoporosis specify that management should be guided by an assessment of patients’ absolute risk of fractures [1]. Recommendations from these guidelines include, obtaining a history and physical examination to identify risk factors for falls and fractures that would warrant further radiographic imaging, bone mineral density (BMD testing), and calculation of 10-year risk of major osteoporotic fractures (i.e., fracture of the hip, clinical vertebra, forearm or proximal humerus). Fracture risk assessment can be conducted with either the Fracture Risk Assessment tool (FRAX®) [6] or the Canadian Association of Radiologists and Osteoporosis Canada (CAROC) absolute fracture risk assessment [7]. Those deemed at high risk should be considered for both pharmacological (antiresorptive agents, bone-forming agents, calcium and vitamin D supplementation) and non-pharmacological (exercise, falls prevention, smoking cessation) interventions [1]. The use of FRAX® has been included in many osteoporosis CPGs to facilitate osteoporosis case-finding and treatment decisions [1, 8–10]. Despite the availability of fracture risk assessment tools and advances in pharmacological therapies, osteoporosis continues to be underdiagnosed and undertreated [11–13]. Barriers to the use of osteoporosis CPG include clinician uncertainty about fracture risk assessment, lack of clinical protocols and organizational processes to support use of best practices [11, 14, 15], concerns about medication side effects [16, 17], and lack of knowledge on medication use (drug holidays, when to stop) [11]. Fracture liaison services have been demonstrated to improve communication, management, and outcomes; however, these discharge coordination initiatives are rare in Canadian hospitals [18]. Much of the research on improving treatment in osteoporosis has focused on interventions in specialized fracture clinics [18], but very little has been conducted at the primary care level. Family physicians have identified a need for clinical support tools that identify potential risk factors, calculate fracture risk, and advice on treatment options [19]. In a study of 1054 family physicians in Ontario, 77% identified the lack of electronic medical record (EMR) tools as a significant barrier to implementing the osteoporosis CPG [20]. The absence of EMR reminders and recalls for medications requiring regularly scheduled injections has been identified as decreasing adherence and causing discontinuation [11].

There is evidence in the literature that clinical decision support systems that integrate decision support tools within EMR software can improve care processes and health outcomes [21–23]. A review of research on EMR dashboards found that they provide a significant opportunity for efficiently and accurately gathering and processing patient data and improving quality of care [24]. A proof of concept study on an EMR quality dashboard focused on 17 health care clinical indicators, not including osteoporosis, found that within 90 days of training, documentation (coding) of patient diagnoses (diabetes, hypertension), screening results (colorectal cancer, breast cancer), and smoking and body mass index status increased by up to 4% [25]. Similar quality improvements were found with the use of a diabetes-specific EMR dashboard [26]. Consistent with these types of dashboards and building on the identified needs of Canadian family physicians for more education on the assessment and management of osteoporosis and their desire for EMR-based tools to support guideline implementation [20], we developed the D**a**shboar**d** Initiati**v**e for Qu**a**lity Improveme**nt** in the M**a**na**g**ement of Pati**e**nts with **O**steo**p**orosis (ADVANTAGE OP). The ADVANTAGE OP dashboard serves as a mechanism for alerting physicians of patients’ potential fracture risk to improve screening and management. In this study, we used this EMR-based osteoporosis decision-support tool to identify potential care gaps in the implementation of best practice recommendations in patients with or at risk for fracture and assess the management of these patients by Canadian primary care physicians.

## Methods

### Participants

A convenience sample of two-hundred family physicians practicing across Canada whose practice used Telus PS Suite and MedAccess EMR platforms were invited to participate in this study. Telus also advertised recruitment for this study in their corporate newsletter and other educational forums. The most common EMR vendor is Telus EMR platforms in Ontario and to a lesser extent British Columbia and Alberta. Participants were required to use the ADVANTAGE OP EMR dashboard for a period of 18 months (July 31^st^, 2018–December 31, 2019) to share practice level EMR data (not patient level data) and where relevant provide the reasons why management differed from that recommended in Canadian CPGs. At the end of the 18-month time period, participants’ practice level data were downloaded for analysis. Participants received monetary compensation (fair market value honorarium) to use the ADVANTAGE OP dashboard to review their practice-related osteoporosis assessment and management; it was anticipated that total time commitment in this study would be a minimum of 12 h.

### Electronic medical record dashboard

Participants completed an educational session, which included an introduction to the osteoporosis and falls custom EMR tool [27] and instruction on how to use the ADVANTAGE OP EMR dashboard. Participants also had access to a study specific protocol that contained educational resources on risk assessment and osteoporosis management, including Canadian CPGs. The ADVANTGE OP EMR dashboard is an interactive algorithm designed to facilitate the use of evidence-based management strategies related to: (1) BMD testing for patients potentially at high risk, (2) fracture risk assessment, and (3) appropriate utilization of pharmacological therapy based on Canadian osteoporosis CPGs [1]. Consistent with the Canadian osteoporosis CPGs, patients were deemed as potentially at high risk for fracture when any of the following criteria are met: (1) all females, age ≥70 years, (2) age ≥50 years with any of the following criteria documented: fracture of the proximal humerus, wrist, vertebral, pelvis, or femur, or, (3) femoral neck or lumbar spine t-score less than or equal to −2.5 [7].

**Fig. 1:**
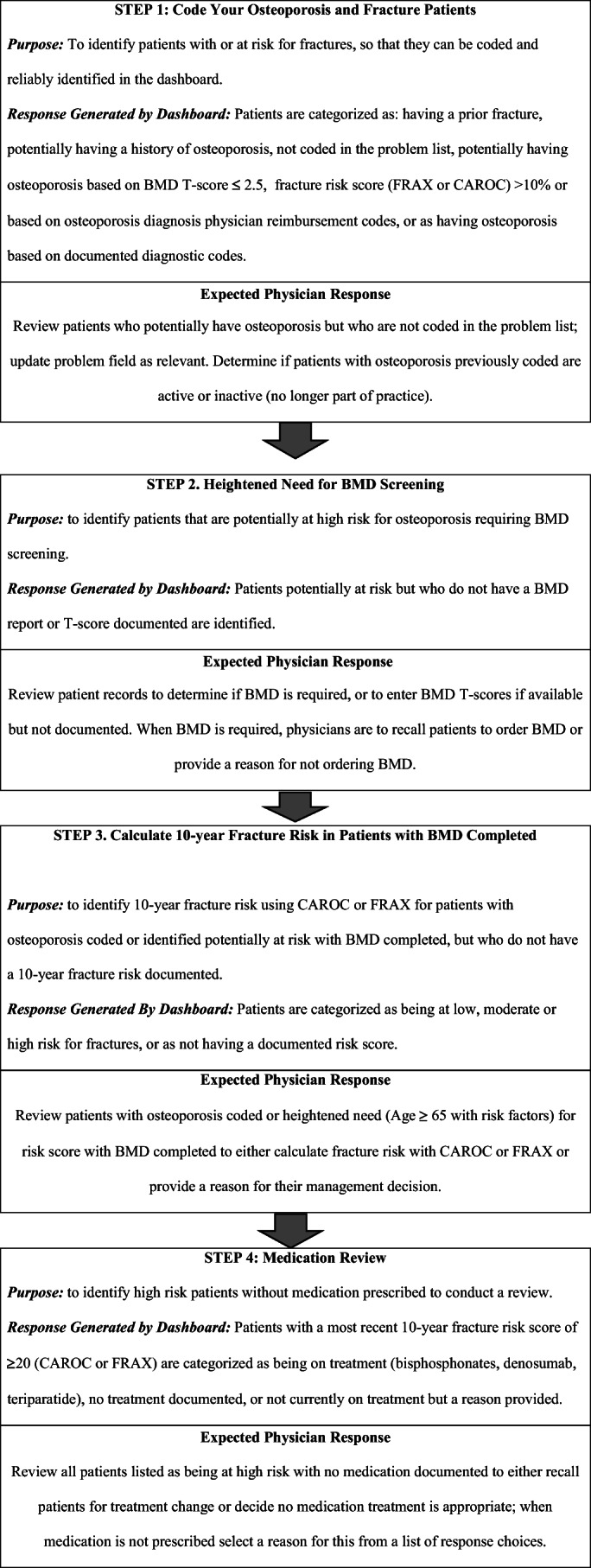
Summary description of the ADVANTAGE OP electronic medical record dashboard CAROC, Canadian Association of Radiologists and Osteoporosis Canada; FRAX®, Fracture Risk Assessment tool

The EMR dashboard consisted of four steps that users proceeded through; these steps are summarized in Fig. 1. Within the EMR dashboard, patients within each participants’ practice with osteoporosis and fractures were identified based on Systematized Nomenclature of Medicine Clinical Terms (SNOMEDCT) code 64859006 or International Classification of Disease 9 (ICD-9) codes for fracture or osteoporosis [28], BMD T-scores (≤2.5), high 10-year fracture risk according to FRAX® (≥20% or ≥3% hip fracture risk) or CAROC (Step 1). To ensure all relevant patients were included in the dataset, those patients who had a prior fracture or potentially having a history of osteoporosis, but who did not have this listed in the EMR problem list were identified for physicians to determine if they met ICD-9 criteria for fracture or osteoporosis codes. Participants were asked whether they would like to request BMD testing for patients potentially at high risk for fractures, including those at high risk who are on treatment and require annual BMD testing and those at moderate risk on treatment who require BMD testing every three years. When it was decided not to order BMD testing, participants were asked to provide a reason for their management decision from the following: patient refusal, not easily accessible/available in my practice area, prior BMD was normal/low risk, do not believe BMD is required, would like specialist referral for BMD, referred for BMD/awaiting results, or, will refer for BMD soon. Physicians were able to select more than one reason for why BMD was not ordered (Step 2). Patients with available BMD test results but for whom 10-year risk of fracture was not yet calculated were identified and for each patient, participants were asked to calculate the individual’s fracture risk using FRAX® or CAROC, both of which are embedded in the dashboard (Step 3). Patients identified at high risk for fracture based on fracture risk calculations with no prescribed fracture prevention medications (e.g., bisphosphonates, denosumab, or teriparatide) were identified and participants were asked if they would treat them based on CPGs and if not, provide a reason for their management decision from a specified list of reasons: patient refusal, social reason (cost, access), medical reason (side effects, intolerance, comorbid conditions), specialist prescribed treatment plan, belief that no treatment was the most appropriate plan, disagreement with recommendation, lack of comfort with making treatment changes, or, intentions to modify the treatment plan (Step 4). Physicians were able to select more than one reason for this management decision. The list of therapies in the dashboard was complete for those medications available based on provincial drug plans and most frequently used in Canada for fracture prevention.

### Outcome measures

The primary outcome measures were: (1) proportion of patients potentially at high risk for fractures based on study criteria for whom BMD testing was not completed and reasons why, (2) proportion of patients for whom BMD testing was completed but T-score was not documented, and (3) the proportion of patients at known high risk for fractures based on CAROC or FRAX® who were not treated according the osteoporosis CPG [1].

### Statistical analysis

Patient counts for each participant were calculated automatically within the Telus system for each of the four steps within the dashboard and downloaded as aggregated across all participants in an excel.csv format. Descriptive analysis of each metric was reported using frequency and percentage. All analysis was performed using SAS 9.4 software (SAS Institute, NC, USA).

## Results

A total of 154 physicians agreed to participate in this study; 84 (54.5%) shared their practice activities using the dashboard. Of the participants who shared their practice data, the majority were from Ontario (*N* = 51; 60.7%). The remaining participants were from British Columbia (*N* = 17; 20.2%), Alberta (*N* = 9; 10.7%), Nova Scotia (*N* = 6; 7.1%) and Saskatchewan (*N* = 1; 1.2%).

**Fig. 2:**
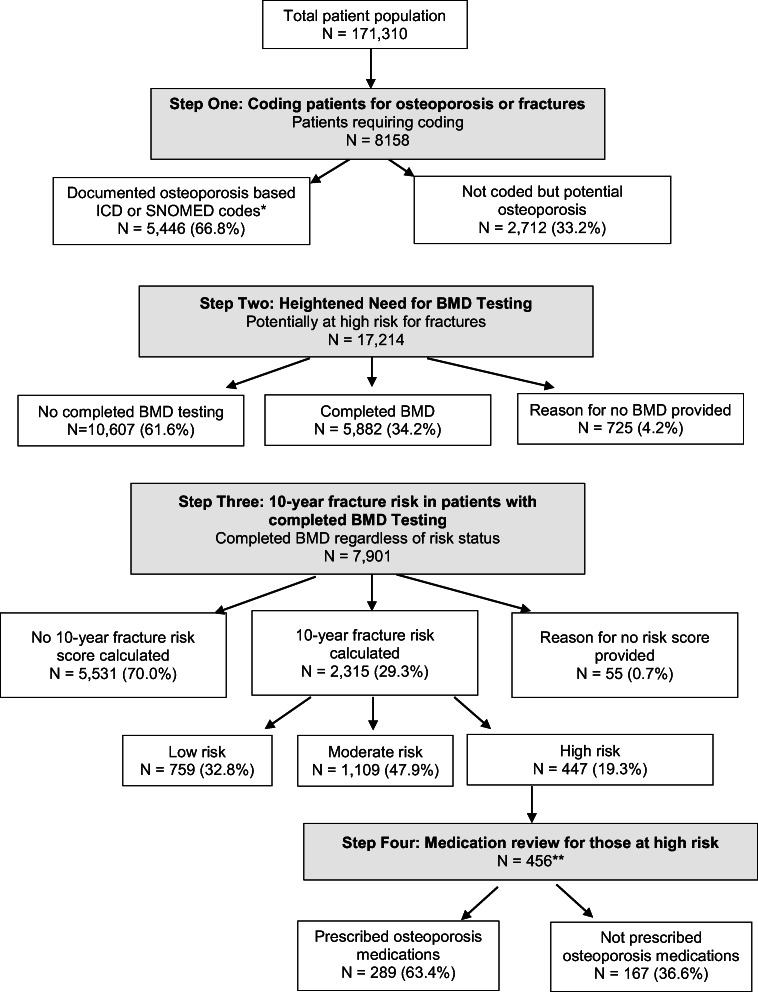
Summary flow chart of the dashboard results. BMD, Bone Mineral Density

A summary flow chart of the dashboard results is presented in Fig. 2. Among the total patient population (171,310) across all participants (*N* = 84), 8,158 patients were identified in Step 1 as needing to be coded with osteoporosis or fracture in the EMR problem list; 66.8% (*N* = 5,446) of whom had osteoporosis based on documented ICD-9 733 or with SNOMEDCT code 64859006. Physicians were asked to review the files of 2,712 (33.2%) patients, who potentially could have osteoporosis but who were not coded as such. Some of these patients (17.8%, *n* = 482) had risk scores (based on FRAX® or CAROC) of equal to or greater than 20% but were not coded as having osteoporosis. Others had BMD T-scores ≤2.5 (2.8%, *n* = 75), a diagnosis of osteoporosis listed in two or more physician reimbursement codes (23.9%, *n* = 648), or osteoporosis documented as text (23.9%, *n* = 647) but were not coded as having osteoporosis. Similarly, 31.7% of patients (*n* = 860) had a fracture documented as text, but were not coded as having had a fracture.

### Heightened need for BMD testing

**Fig. 3:**
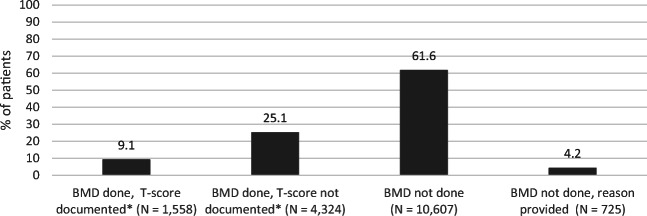
Status of BMD screening among patients identified potentially at risk for fractures (*N* = 17,214)

**Fig. 4:**
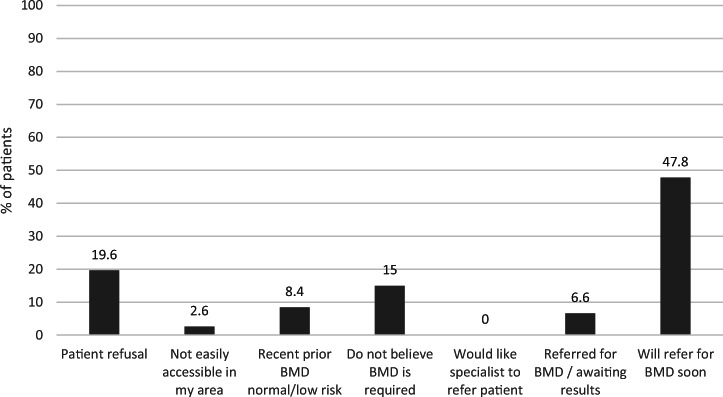
Barriers for ordering BMD for patients potentially at risk (*N* = 1065)*

**Fig. 5:**
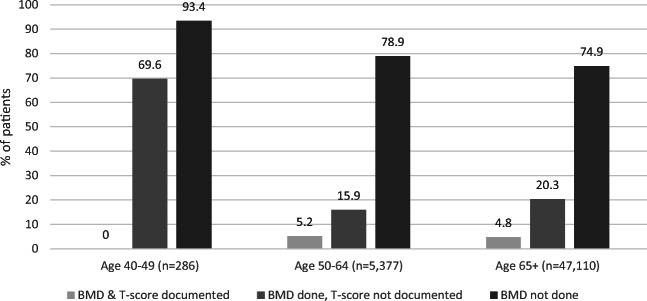
Need for BMD screening among all patients at risk for osteoporosis by age (*N* = 55,763)

Among the whole patient population (171,310), a total of 17,214 (10%) patients were identified as potentially at high risk for fractures, the majority of whom (61.6%, *n* = 10,607) did not have completed BMD testing whereas 34.2% (*n* = 5,882) did (Figs. 2 and 3). Among patients potentially at high risk for fracture with completed BMD testing (*N* = 5,882), only 26.5% (n = 1,558) of patients had their T-scores documented within their EMR. Of those patients identified as potentially at risk for fractures (N = 17,214), a relatively small proportion (4.2%, *n* = 725) had a reason listed for why BMD testing was not completed. Fig. 4 presents the reasons physicians provided for why BMD testing was not completed for patients potentially at high risk; the most common being patient refusal in 19.6% (*n* = 209) and 47.8% (*n* = 509) indicating intentions to order BMD testing. BMD testing by age is shown in Fig. 5. In almost 75% (*n* = 35,308) of patients 65 years of age and older, BMD testing was not completed.

### 10-year Fracture risk in patients with BMD

Among all patients with completed BMD screening (*N* = 7901; regardless of risk status for fractures), risk scores were calculated using FRAX® or CAROC in 29.3% (*n* = 2,315) of patients; 70.0% (*n* = 5,531) of patients did not have risk scores calculated and 0.7% (*n* = 55) had a reason listed for why a fracture risk score was not calculated. Among those with calculated risk scores (*N* = 2,315), 19.3% (*n* = 447) were at high fracture risk; 32.8% (*n* = 759) were at low risk and 47.9% (*n* = 1,109) were at moderate risk.

### Medication review

**Fig. 6:**
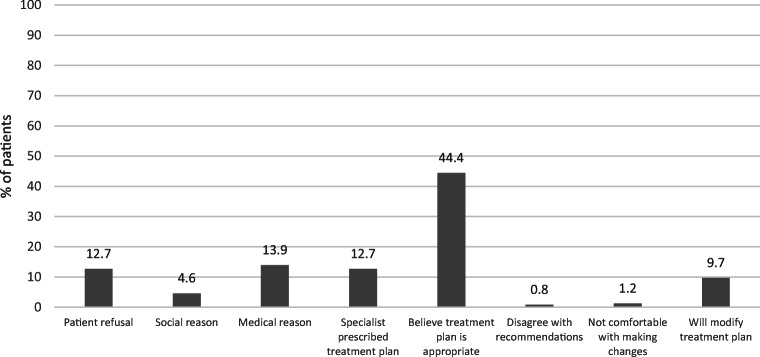
Reasons provided for non-treatment in high risk patients (*N* = 259)*

Among those at high fracture risk (*N* = 456), 63.4% (*n* = 289) were on medications for osteoporosis (*n* = 167 on bisphosphonates; *n* = 122 on denosumab). The most common reason for non-treatment was the belief that this was appropriate (44.4%, *n* = 115); in 9.7% of cases (*n* = 25), the physician intended to modify the treatment plan (Fig. 6). Other reasons included patient refusal, social or medical reasons, a specialist made treatment decisions, disagreement with recommendations and lack of comfort in making treatment changes.

## Discussion

Despite evidence that interventions combining education and EMR-based alerting systems can increase BMD screening and appropriate osteoporosis medication prescribing [29], in this study, there continued to be a significant number of patients at risk who were not screened or treated.

Point-of-practice tools including algorithms and checklists, and integration of decision support tools into EMRs have been identified as strategies for supporting CPG implementation [30]. Alerting systems appear to be key to facilitating practice change. A study examining the use of an osteoporosis order set embedded within an EMR found that while it increased the prescribing of calcium, it had minimal impact on the prescribing of osteoporosis medication; these findings were attributed, in part, to the lack of an alerting or reminder function [31]. While physicians value point-of-practice tools and have identified a desire more EMR-based tools to facilitate clinical decision making, the fact exists that these tools create an extra layer to their work flow. Tool complexity, lack of time to become familiar with and use the tool during patient visits, and financial disincentives have been identified as barriers to using these types of tools in clinical practice [20]. In this study, fracture risk scores were only calculated in 29% of patients with completed BMD screening. In a qualitative study of physicians’ views of osteoporosis management, physicians reported that their limited use of the FRAX® tool for calculating fracture risk was because the FRAX® was not embedded into the EMR [32]. Intuitively it makes sense that the extra step of accessing FRAX® online would be barrier; however, in the current study, FRAX® and CAROC were embedded within the dashboard for immediate access. Other strategies are needed to facilitate their use. The development of tools that self-populate with existing information within the EMR (passive data collection) may address barriers related to time constraints and the performance of extra steps to ascertain results to better identify those at risk for fractures [33, 34]. Moreover, it has been suggested that combined education and decision support tools aimed at both physicians and patients may be effective for osteoporosis management [35].

This study highlights significant care gaps in the assessment and management of osteoporosis, despite the use of an EMR-based dashboard to improve treatment. Only a third of those potentially at high risk for fractures had BMD testing completed, and among all patients with BMD completed, only a third had 10-year fracture risk scores documented. Among those with high 10-year fracture risk scores, a third were not on treatment. These findings are consistent with other studies that have found that, despite the prevalence of fragility fractures, there remains a considerable care gap [2, 3, 36, 37]. The lack of treatment following a fracture is in sharp contrast to other conditions where one medical event is predictive of subsequent events, such as acute myocardial infarction after which 96% of patients are prescribed beta-blockers and 98% aspirin to prevent subsequent events [38]. We found that a high proportion of physicians indicated intentions to order BMD testing “soon”, the fact remains that they did not order the testing when first alerted to potential risk. When physicians did order BMD testing, the resulting T-scores were often not documented, preventing a potential alert for calculating fracture risk. Challenges into the integration and usability into workflow included osteoporosis and fractures were not well coded/documented in the EMR with ICD codes that identify fractures and osteoporosis, and BMD reports not automatically populating the EMR [39].

Forty-four percent of physicians indicated that they were not prescribing medication to patients at high risk of fracture because they believed their current care plan for their patients was appropriate. It is not clear why physicians perceive a treatment plan contrary to CPG would be appropriate. Potential adverse effects, particularly as related to potential atypical femur fractures associated with bisphosphonate use, scepticism about treatment efficacy, and a perceived lack of urgency for treating osteoporosis have recently been identified as reasons for not prescribing osteoporosis medications [11, 12, 16]. Further research is needed to better understand why physicians choose not to treat patients for osteoporosis and how this might best be mediated, particularly with education aimed at addressing negative physician attitudes and beliefs towards osteoporosis treatment. There may be factors related to comorbid conditions, such as a desire to minimize polypharmacy or physician attitudes towards osteoporosis and fracture prevention. There is some evidence that physicians believe that osteoporosis is not as much of a priority to treat in comparison to other conditions such as diabetes, cardiovascular disease and hypertension [32, 40]. Organizational support may be an additional factor facilitating the implementation CPG and point-of-practice tools [41–43]. As physicians in this current study were recruited individually, not at a practice setting level, the role of organizational support and promotion in using the Advantage OP dashboard is not known. Further research is needed to understand the role that organizational support and environmental context play in facilitating the use of the dashboard.

### Limitations

Participants in this study were self-selected and thus selection bias may impact the generalizability of our findings. The dashboard did not include raloxifene and hormone therapy as anti-fracture therapies, which may have resulted in an underestimation of patients receiving some fracture protection. However, while these medications are both available in Canada, utilization in Canada and other countries for osteoporosis is low and thus it was decided not to include them in the dashboard [44, 45]. As practice level data were collected, the absence of patient level data limits our understanding of the decisions made about the BMD screening and treatment. While 447 patients were deemed at high risk for fractures based on 10-year fracture risk calculations, information on the management of 456 patients was generated. At the time that physicians shared their practice information at study end, there were 25 cases in which they intended to modify the patients’ treatment plan; information on whether this occurred is not available. In total, 7,901 patients had BMD testing completed regardless of risk status. While it is not entirely clear why patients at low risk were completing BMD testing and some at high risk were not being tested, this could reflect that some patients may have completed BMD testing over the age of 65 who were not at risk, consistent with CPGs [1]. The collection of practice level data also does not allow us to fully understand changes in observations, which may result when patients leave and new patients enter a practice or their health status changes as classified by their physician. A high proportion of participants (46%) did not share their practice data. It has been suggested that this might be due to perceptions that it involved too much time for the amount of work and compensation provided or, because this was new learning for some physicians, they were unsure of how to “share” data and may have been uncomfortable with sharing their data. It has also been suggested that as use of the dashboard requires a staff member to execute patient recalls, limited health human resources may have limited use of the dashboard. Following the study time period, we surveyed participants to gather information about their practice model and to assess their perceptions of their use of this tool including perceived helpfulness in calculating 10-year fracture risk and assessing and managing patients at heightened risk for fractures, feasibility of use, identification of potential barriers to utilizing the tool as part of routine clinical practice and likelihood of continued use of the tool. In particular, we were interested in identifying the barriers to use by those physicians who had initially agreed to participate but failed to use the tool. Unfortunately, the response rate to this survey was less than 10%, so we are unable to provide a greater understanding of the continued clinical inertia in osteoporosis diagnosis and management. We attempted to gather some of this information as part of presentations that we conducted to share study results with participants. A facilitated discussion followed, asking for facilitators and barriers to treatment of osteoporosis. Identified facilitating factors included having the guidelines embedded in the EMR tool and including questions on risk factors for FRAX®. Barriers included the following: having to input bone mineral density values into the EMR form, the EMR tool not automatically calculating the FRAX® score, and not being familiar with FRAX® as the majority of radiologists report using CAROC.

Future studies will need to better understand the barriers to using this type of clinical practice tool and potential changes and strategies needed to improve use of the dashboard. For example, there may be opportunities to increase use of the dashboard by involving primary care nurses in its use. Nurses could maintain documentation on patients experiencing a fracture or having been diagnosed with osteoporosis (Step 1 in the dashboard process), flagging those requiring BMD testing (Step 2) and then flagging those requiring fracture risk assessment to family physicians.

This was an observational study. More rigorous methodologies, including randomized controlled trials comparing the use of the Advantage OP dashboard with usual care will increase our understanding of the impact associated with this EMR tool. Further research is needed to better understand perceptions of the Advantage OP dashboard and opportunities to improve and sustain usage.

## Conclusion

Despite the use of an EMR decision support tool for osteoporosis, there continues to be significant gaps in BMD testing, fracture risk calculation and management among high risk patients, suggesting that additional knowledge translation strategies are needed to improve both osteoporosis screening and care. A better understanding of physician interface with EMR decision support tools as well as patient decision-making is needed to inform the development of strategies and tools that will better support the implementation of osteoporosis CPG. Point-of-practice and decision support tools, including those embedded within EMR platforms, should be utilized to address existing knowledge and action (practice) gaps and will need to include functions beyond alerting and reminders to support CPG implementation.

## Data Availability

Available upon request.
